# Driving pressure during proportional assist ventilation: an observational study

**DOI:** 10.1186/s13613-018-0477-4

**Published:** 2019-01-03

**Authors:** Katerina Vaporidi, Charalambos Psarologakis, Athanasia Proklou, Emmanouil Pediaditis, Evangelia Akoumianaki, Elisavet Koutsiana, Achilleas Chytas, Ioanna Chouvarda, Eumorfia Kondili, Dimitris Georgopoulos

**Affiliations:** 1 Department of Intensive Care Medicine, University Hospital of Heraklion, School of Medicine, University of Crete, Voutes, 71110 Heraklion, Crete Greece; 2 Lab of Computing Medical Informatics and Biomedical Imaging Technologies, School of Medicine, Aristotle University of Thessaloniki, Thessaloníki, Greece; 3 Institute of Applied Biosciences, CERTH, Thessaloniki, Greece

**Keywords:** Protective ventilation, Compliance, Tidal volume, Monitoring

## Abstract

**Background:**

During passive mechanical ventilation, the driving pressure of the respiratory system is an important mediator of ventilator-induced lung injury. Monitoring of driving pressure during assisted ventilation, similar to controlled ventilation, could be a tool to identify patients at risk of ventilator-induced lung injury. The aim of this study was to describe driving pressure over time and to identify whether and when high driving pressure occurs in critically ill patients during assisted ventilation.

**Methods:**

Sixty-two patients fulfilling criteria for assisted ventilation were prospectively studied. Patients were included when the treating physician selected proportional assist ventilation (PAV+), a mode that estimates respiratory system compliance. In these patients, continuous recordings of all ventilator parameters were obtained for up to 72 h. Driving pressure was calculated as tidal volume-to-respiratory system compliance ratio. The distribution of driving pressure and tidal volume values over time was examined, and periods of sustained high driving pressure (≥ 15 cmH_2_O) and of stable compliance were identified and analyzed.

**Results:**

The analysis included 3200 h of ventilation, consisting of 8.8 million samples. For most (95%) of the time, driving pressure was < 15 cmH_2_O and tidal volume < 11 mL/kg (of ideal body weight). In most patients, high driving pressure was observed for short periods of time (median 2.5 min). Prolonged periods of high driving pressure were observed in five patients (8%). During the 661 periods of stable compliance, high driving pressure combined with a tidal volume ≥ 8 mL/kg was observed only in 11 cases (1.6%) pertaining to four patients. High driving pressure occurred almost exclusively when respiratory system compliance was low, and compliance above 30 mL/cmH_2_O excluded the presence of high driving pressure with 90% sensitivity and specificity.

**Conclusions:**

In critically ill patients fulfilling criteria for assisted ventilation, and ventilated in PAV+ mode, sustained high driving pressure occurred in a small, yet not negligible number of patients. The presence of sustained high driving pressure was not associated with high tidal volume, but occurred almost exclusively when compliance was below 30 mL/cmH_2_O.

**Electronic supplementary material:**

The online version of this article (10.1186/s13613-018-0477-4) contains supplementary material, which is available to authorized users.

## Background

The driving pressure of respiratory system (Δ*P*) during passive mechanical ventilation is defined as the difference between static end-inspiratory plateau pressure (Pplat) and static positive end-expiratory pressure (PEEP) and equals the ratio of tidal volume (*V*_T_) to respiratory system compliance (Crs). Therefore, Δ*P* reflects the extent of lung stretch at end inspiration better than *V*_T_ alone (when *V*_T_ is set), because it takes into account patient’s respiratory system compliance. Despite the fact that Δ*P* represents a global measurement of lung stretch and thus cannot capture lung inhomogeneity, recent studies have shown that Δ*P* is a main determinant of ventilator-induced lung injury (VILI), and it is associated with mortality in ARDS patients, particularly at Δ*P* values above 14 cmH_2_O [1–6]. In addition, even in patients with uninjured lungs, an association between high Δ*P* and increased morbidity has been postulated [4, 7, 8].

Although Δ*P* as a risk factor for VILI has been exclusively studied in patients under controlled mechanical ventilation, the potentially harmful effects of high Δ*P* are probably present in any mode of ventilation. Recently, the concept of self-inflicted lung injury has been introduced, referring to patients in assisted ventilation [9–11]. As the beneficial effects of spontaneous breathing during mechanical ventilation are well established, it becomes increasingly important to identify patients at risk of self-inflicted lung injury during assisted ventilation [9–11]. To identify patients at risk and prevent self-inflicted lung injury, monitoring of Δ*P* during assisted ventilation might be helpful. Experimental data indicate that during assisted ventilation vigorous spontaneous efforts increase transpulmonary driving pressure and worsen lung injury [12, 13]. However, limited information is available on the presence of high Δ*P* in patients ventilated in assisted modes, mainly because measuring Δ*P* requires valid estimation of Crs, a complicated task with conventional assisted modes of ventilation such as volume assist and pressure support [14].

In a recent study [15], we have reported data on Δ*P* obtained using proportional assist ventilation with load-adjustable gain factors (PAV+), a mode validated to measure end-inspiratory quasi-static Pplat, and compute Crs [16–19]. In this study, using single measurements of Δ*P* obtained when patients were switched from controlled ventilation to PAV+, we found that Δ*P* was mostly below 15 cmH_2_O, while *V*_T_ was usually higher than that set during controlled ventilation [15]. Nevertheless, because in spontaneously breathing patients there is considerable variability in breathing patterns, prolonged and continuous measurements of Δ*P* would be required to fully capture the spectrum of Δ*P* during assisted ventilation.

In the current study, we described Δ*P* over time, aiming to explore whether and when high Δ*P* occurs in everyday clinical practice in patients placed in assisted ventilation, using continuous measurements of Δ*P* obtained in PAV+ mode. We hypothesized that sustained high Δ*P* (≥ 15 cmH_2_O), and hence increased risk of injury, would be present during periods of relative hyperventilation, when tidal volume and minute ventilation would be high, and/or during periods when Crs would be relatively low, and sought to identify potential safe thresholds for *V*_T_ and/or Crs.

## Materials and methods

### Design and setting

This study was conducted in a medical–surgical intensive care unit (ICU). The study was approved by the Hospital Ethics Committee, and since there was no interference with patients’ management, signed informed consent was waived. A detailed description of methods is presented in Additional file 1. Patients were included at any time the treating physician placed them in PAV+ mode and estimated that they would remain on assisted mechanical ventilation for more than 1 day. Patients were excluded if the level of assist in PAV+, as chosen by the primary physician, was less than 20%, or when the necessary equipment for the recording was unavailable. The recording period was 72 h unless the patient was placed on T-piece earlier. During the recording period, treating physicians could change ventilator mode at their best judgment. The ventilator was connected to a bedside computer, and a continuous recording of all ventilator parameters was obtained at a frequency of 0.8 Hz using dedicated software.

### Data analysis

**Fig. 1:**
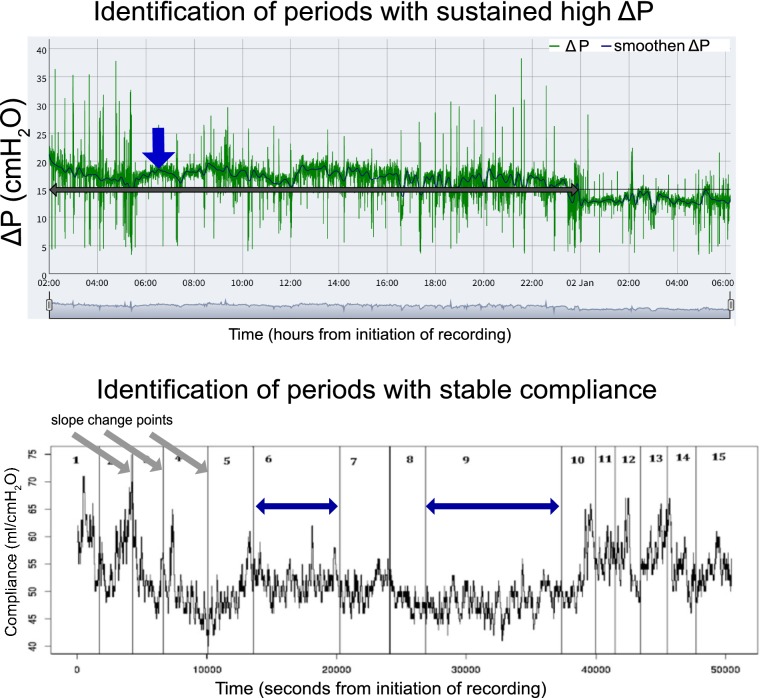
Upper panel: Identification of periods of sustained high Δ*P*: representative plot from one patient, generated via an R-Shiny application built for this purpose, showing the driving pressure (Δ*P*) time-series signal, raw data in green, and smoothen signal in blue (marked with thick blue arrow). The thick horizontal gray arrow indicates the period of sustained high Δ*P* ≥ 15 cmH_2_O. In* x*-axis is time in hours from initiation of recording. Lower panel: Identification of periods of stable compliance: representative plot of one patient showing compliance over time (*x*-axis showing time in seconds from initiation of recording). In the first step of this analysis, the slope change points (gray arrows) are identified, in the second step, the slope of each segment is calculated (segments numbered here from 1 to 15), and in the third step, periods with slope between − 0.001 and 0.001 are selected as periods of stable compliance (in this case periods 6 and 9 shown with blue arrows)

A more detailed description of the analysis is presented in Additional file 1. The recordings were processed before analysis to optimize data quality (e.g., artifact rejection) and exclude the measurements obtained in other modes of ventilation (if there was a change in mode during the 72-h period). Δ*P* was calculated from the measurements of respiratory system compliance (Crs) and tidal volume (*V*_T_) as Δ*P* = *V*_T_/Crs, as described in detail in additional file 1. (In this calculation, PEEPi was not taken into consideration.) Three types of analysis were performed (see also Additional file 1). First, the distribution of Δ*P* and *V*_T_ values over time was calculated using raw data. Second, periods of high Δ*P* sustained for more than 1 h were identified after a smoothing was applied to the Δ*P* signal (Fig. 1). A time frame of at least 1 h was chosen so that possible correlations with the hourly collected data on vital signs and medication could be explored. Lastly, periods of stable compliance were identified after analyzing the slope of the Crs signal (Fig. 1). For the entire recording during PAV+, and for all selected periods (high Δ*P*, stable Crs periods), the mean, median, standard deviation, and interquartile range for all set and measured parameters of the ventilator were calculated using R programming language and software environment.

### Statistical analysis

Continuous variables are reported as means and standard deviation (SD) for normally distributed data and as medians and interquartile ranges (IQR) for non-normally distributed data. Categorical variables are presented as percentages. Between-group differences in continuous variables were compared using Mann–Whitney U test. Differences in ventilation or clinical parameters in the same patient between different periods were compared with Friedman’s two-way analysis of variance by ranks. Spearman’s rho was used to evaluate correlations between continuous variables. A* p* value of < 0.05 was considered significant. We used IBM SPSS Statistics for Windows version 22 (Armonk, NY) for analysis.

## Results

We obtained demographic, clinical, and ventilation data from 62 patients during a 2-year period. During the same period, 617 patients were admitted in the ICU and remained on mechanical ventilation (any mode) for more than 48 h. Overall, 8.8 million samples corresponding to 3200 h of ventilation were analyzed. Patients’ characteristics are presented in Table 1. Patients at time of inclusion had been on mechanical ventilation for a median of 7 days (IQR = 4–12 days) and remained on mechanical ventilation after the beginning of the recording for another 7 days (IQR = 3–15 days). Most patients (95%) were receiving antibiotics for suspected or confirmed ICU-acquired infections. Thirty-five patients (56%) fulfilled criteria of Berlin definition for mild or moderate ARDS, which, in most cases, was associated with the ICU-acquired infection. The median ICU stay was 22 days (IQR = 14–32 days), the total duration of mechanical ventilation was 18 days (IQR = 12–25 days), and the ICU mortality was 45%.

**Table 1:** Patients’ Characteristics

Demographics	Total *n* = 62
Male % (*n*)	68 (42)
Age (mean, SD)	65 ± 16
BMI (median, IQR)	28.4 (26–33.7)
Severity Scores on admission (mean, SD)	
APACHE-II	25 ± 7
SOFA	10 ± 3
Admission diagnosis^1^ % (*n*)	
Sepsis	35 (22)
Multiple Trauma	16 (10)
CNS injury^2^	21 (13)
Postoperative	11 (7)
Pneumonia/LRTI	23 (14)
Other	16 (10)
ARDS present on admission	45 (28)
Ventilation characteristics at inclusion	
Tidal volume, mL/kg IBW^3^	6.6 (5.8–7.8)
PAV+ % assist	50 (40–50)
PEEP	7 (6–9.5)
PO_2_/FiO_2_	200 (167–246)
Tracheostomy present % (n)	44 (27)
Clinical characteristics at inclusion	
SOFA score (mean ± SD)	8 ± 3
Mild ARDS % (*n*)	34 (21)
Moderate ARDS % (*n*)	22 (14)
Metabolic acidosis % (*n*)	61 (38)
Norepinephrine > 0.1 μg/kg/min % (*n*)	5 (3)
Antibiotics^4^ % (*n*)	95 (59)
Sedation (propofol and/or midazolam, any dose) % (*n*)	13 (8)
Opioid analgesics (any dose) % (*n*)	53 (33)
Remifentanil or fentanyl dose (in mg/h, median, IQR)	0.2 (0.1–0.3)

*BMI* body mass index (kg/m^2^),* APACHE-II* Acute Physiology and Chronic Health Evaluation II,* SOFA* Sequential Organ Failure Assessment,* COPD* chronic obstructive pulmonary disease,* CNS* central nervous system,* LRTI* lower respiratory tract infection,* IBW* ideal body weight,* PAV+* proportional assist ventilation,* PEEP* positive end-expiratory pressure,* ARDS* acute respiratory distress syndrome, according to Berlin definition

^1^Admission diagnosis: more than one may apply in each patient

^2^CNS injury traumatic and non-traumatic

^3^Tidal volume, PEEP, and PO_2_/FiO_2_ just before inclusion

^4^Antibiotics were administered for suspected or confirmed ICU-acquired infection

### Analysis of driving pressure and tidal volume over time

**Fig. 2:**
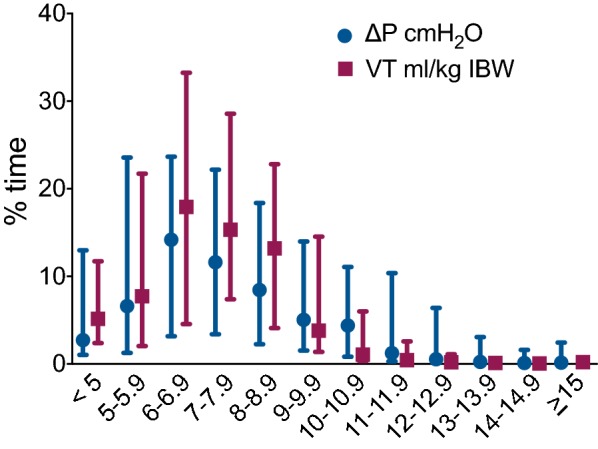
Time as  % of the total analyzed period, with Δ*P* and *V*_T_ values within the range of each cmH_2_O or mL/kg of ideal body weight, from less than 5 to more than 15, expressed as median and interquartile range of all patients’ values

The median analyzed period (recording time free of artifacts and in PAV+ mode) per patient was 44 h (IQR = 26–72 h). All respiratory variables varied over time in the same patient, and the median coefficient of variation for Crs, Δ*P*, and *V*_T_ was 11.5, 19, and 21%, respectively. Specifically for Δ*P*, the interquartile range during the recording was 2 cmH_2_O, reaching 5 cmH_2_O in several patients. The relative frequencies of Δ*P* and *V*_T_ values during this period were examined by calculating the time that these values were within the range of each cmH_2_O (for Δ*P*) or mL/kg of ideal body weight (for *V*_T_), from less than 5 to more than 15 (Fig. 2). Overall, for most (95%) of the analyzed period, Δ*P* was less than 15 cmH_2_O. The median time with Δ*P* ≥ 15 cmH_2_O was 2.5 min or 0.14% of time (IQR = 0.5–67 min, 0.01–2.4%), but, in five out of 62 patients (8%), a Δ*P* ≥ 15 cmH_2_O was present for more than 12 h (10% of time). The clinical and ventilation characteristics of these patients are presented in Additional file 2: Table S1. Patients with prolonged high Δ*P* had similar age and severity as the rest of the patients, but higher BMI (median = 36, IQR = 30–38, vs. median = 28, IQR = 26–34, *p* = 0.044). Although not an exclusion criterion, abdominal pathology was not present in any of the patients with prolonged high Δ*P*. (Abdominal pressure was not measured.) Tidal volume, normalized for ideal body weight, was not different from the rest of the patients. Overall, there was no significant correlation between BMI and median Δ*P* (cor. coef. = 0.1, *p* = 0.4), and the time with Δ*P* above 15 cmH_2_O was not different between obese and non-obese patients. Moreover, when PEEPi (as measured by the ventilator software, see Additional file 1) was included in the calculation of Δ*P*, the number of patients having prolonged high Δ*P* did not change. To examine the correlation of high Δ*P* with mortality, we compared the time above a pressure threshold between ICU survivors and non-survivors. Survivors (*n* = 34) had less time than non-survivors (*n* = 28) with Δ*P* ≥ 15 cmH_2_O (survivors: median = 1.7 min, IQR = 18 min, non-survivors: median = 10 min, IQR = 115 min, *p* = 0.03), while no difference was observed for lower Δ*P* thresholds (data not shown). Tidal volume was less than 11 mL/kg for 95% of the analyzed time and between 5 and 8 mL/kg for 65% of that time. No difference between survivors and non-survivors was observed for any *V*_T_ threshold.

### Analysis of periods of sustained high driving pressure

Subsequently, periods of high Δ*P* (≥ 15 cmH_2_O) sustained for more than 1 h were identified, aiming to better explore the associations of high Δ*P* with other ventilator variables and clinical characteristics. Eighteen such periods in eight patients were identified. (Five of those patients were also identified having prolonged high Δ*P*.) The median duration of the sustained high Δ*P* periods was 5 h (IQR = 2–9 h). We compared the parameters of ventilation between the high-Δ*P* periods and the rest of the analyzed time in all patients (unpaired comparison) and in each of those patients (paired comparison, Table 2). Periods of high Δ*P* were characterized by higher *V*_T_ and lower Crs. Although statistically significant, the difference of median *V*_T_ between the high and low Δ*P* periods was very small, only 0.3 mL/kg, while the median difference in compliance was 11 mL/cmH_2_O, with no overlapping range. Respiratory rate and minute ventilation were not different between high Δ*P* periods and the rest of the analyzed periods. We also compared clinical parameters between the high Δ*P* periods and the rest of the analyzed time. We did not identify any specific clinical parameter, such as the presence of fever, metabolic acidosis, delirium, opioids, or shock to be related to the presence of high Δ*P* (data not shown). Moreover, arterial PaCO_2_ and pH were not different before and during the high Δ*P* period (median difference of PaCO_2_ = − 0.25 mmHg, *p* = 0.28, and pH 0.011, *p* = 0.07).

**Table 2:** Ventilation parameters during the total analyzed period and the high driving pressure periods (Δ*P* ≥ 15 cmH_2_O)

Ventilation parameters	All patients total analyzed period	18 periods of high Δ*P* versus the rest of the analyzed period (low-Δ*P*) from eight patients	
		Low-Δ*P* period	High-Δ*P* period
FiO_2_(%)	40 (30–50)*	40 (35–50)	40 (35–50)
PEEP (cmH_2_O)	8 (6–8)	8 (8–10)	8 (7–8)
PaO_2_/FiO_2_	239 (182–280)*	246 (174–269)^#^	213 (149–237)
PAV+ % assist	40 (25–50)	40 (25–50)	45 (30–50)
Respiratory Rate (breaths per min)	23 (20–27)	24 (22–28)	25 (22–29)
*V*_T_ mL/kg IBW	7.3 (6.5–8.3)	7.3 (7–7.9)^#^	7.6 (7.3–8.1)
VE (L/min)	10.2 (8.7–11.2)	10.2 (9.1–11.4)	10.1 (8.6–11.9)
R_TOT_(cmH_2_O/L/s)	9.5 (8.3–12)*	10.8 (8.7–13)^#^	12.5 (8.8–15.7)
R_PAV_(cmH_2_O/L/s)	6.2 (4.6–8.6)	7.9 (3.5–9.2)	7.7 (5.8–10.5)
PEEPi (cmH_2_O)	0.3 (0.1–0.7)	0.3 (0–0.5)	0.2 (0.1–0.8)
Crs (mL/cmH_2_O)	56 (42–71)**	38 (32–45)^##^	27 (24–30)
Ti (sec)	0.89 (0.80- 1.04)*	0.85 (0.77–0.97)	0.81 (0.70–0.85)
WOB (J/L)	0.9 (0.8–1.1)**	1.1 (0.9–1.3)^##^	1.5 (1.2–1.7)
Δ*P* (cmH_2_O)	7.8 (6.3–9.9)**	12 (10.2–12.3)^##^	15.6 (15.1–16.1)

*FiO*_2_ fraction of inspired oxygen  %, PEEP: positive end-expiratory pressure,* PaO*_2_ arterial oxygen tension in mmHg, PAV+: proportional assist ventilation, *V*_T_ tidal volume,* VE* minute ventilation,* R*_TOT_ total calculated resistance (patient airways + artificial airway),* R*_PAV_ patient resistance calculated by PAV+ software (difference between* R*_TOT_ and estimated resistance of the artificial airway, after input of intratracheal tube size),* PEEPi* intrinsic PEEP,* C*_PAV_ respiratory system compliance calculated by PAV+,* Ti* inspiratory time,* WOB* work of breathing, Δ*P* driving pressure

Values are presented as median and interquartile range. * *p* < 0.05, ** *p* < 0.0001 for high-Δ*P*-time vs. complete analysis of all patients (Mann–Whitney* U* test), and ^#^*p* < 0.05, ^##^*p* < 0.0001 for high-Δ*P*-time versus low-Δ*P*-time (rest of the recording) of the same patient (Friedman’s two-way analysis of variance by ranks)

### Analysis of periods of stable compliance

**Fig. 3:**
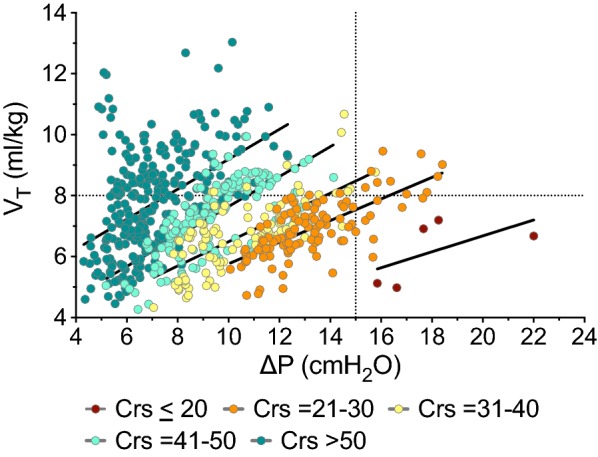
Driving pressure (Δ*P*, in cmH_2_O) vs. tidal volume (*V*_T_, in mL/kg ideal body weight) during all periods of stable compliance (661 periods from 60 patients), colored according to the range of respiratory system compliance (Crs, mL/cmH_2_O). Dotted vertical and horizontal lines indicate thresholds of Δ*P* ≥ 15 cmH_2_O and *V*_T_ ≥ 8 mL/kg, respectively, and solid black lines indicate the correlation lines for each range of compliance. In the upper right quarter (values Δ*P* ≥ 15 cmH_2_O and *V*_T_ ≥ 8 mL/kg), there are 11 points (10 points with compliance 21–30 mL/cmH_2_O and one point with compliance of 31 mL/cmH_2_O), pertaining to four patients

**Fig. 4:**
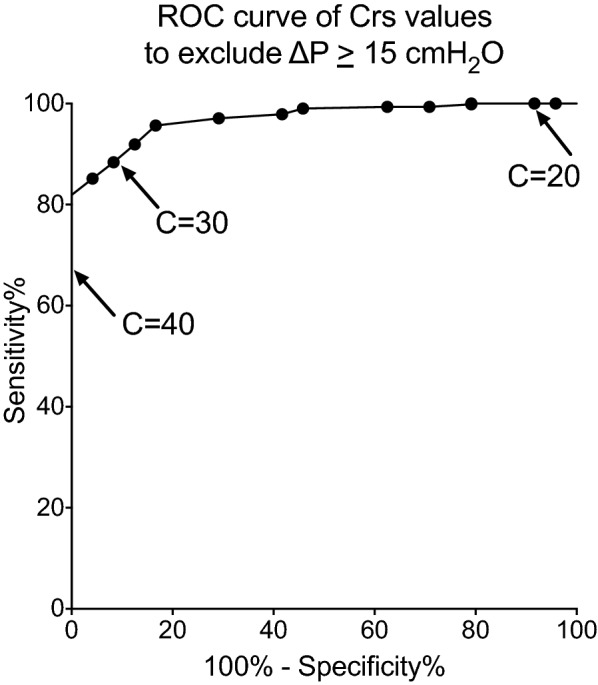
ROC curve for the absence of Δ*P* ≥ 15 cmH_2_O based on compliance, for all periods of stable compliance (661 periods from 60 patients). Arrows indicate the coordinates for the specific values of compliance (AUC = 0.97)

To better explore the correlations of Δ*P* and *V*_T_ across different levels of compliance, all periods of stable compliance were identified, and the corresponding median values of Δ*P* and *V*_T_ were calculated. A total of 661 periods were identified, with a median duration of 125 min (IQR = 58–248 min) and total duration of 2330 h. By plotting the median values of Δ*P* versus *V*_T_ during these periods, we observed that high *V*_T_ was associated with low Δ*P*, and vice versa (Fig. 3). Specifically, Δ*P* values ≥ 15 cmH_2_O, combined with a *V*_T_ ≥ 8 mL/kg, were observed only in 11 cases (1.7% of total periods analyzed), pertaining to four patients. A compliance higher than 30 mL/cmH_2_O was identified as having a sensitivity and a specificity of 90% to exclude the presence of Δ*P* ≥ 15 cmH_2_O (Fig. 4).

## Discussion

This observational study reports, for the first time to our knowledge, continuous and prolonged measurements of driving pressure in everyday clinical practice in critically ill patients during proportional assist ventilation. The main findings of our study are: (1) For most of the analyzed time (95%), driving pressure and tidal volume were below 15 cmH_2_O and 11 mL/kg, respectively. (2) The incidence of prolonged high driving pressure (≥ 15 cmH_2_O) was 8%, and this was not associated with either very high tidal volume (mean 7.5 mL/kg, max. 9.5 mL/kg) or minute ventilation (mean 10 L/min, max. 13 L/min). (3) Independent of tidal volume, episodes of sustained high driving pressure were very unlikely to occur when respiratory system compliance was above 30 mL/cmH_2_O.

Certain methodological issues of the study should be discussed first. To begin with, the measurement of Δ*P* relies on the measurement of compliance used by PAV+ software. Studies have shown that respiratory system mechanics, as measured with PAV+, are similar to those measured during passive mechanical ventilation using standard techniques [16, 17, 20]. Particularly, provided that the level of assist is greater than 20%, Paw measured at 0.3 s from the onset of end-inspiratory occlusion in PAV+ provides a reliable estimate of passive elastic recoil pressure at the corresponding *V*_T_, independent of respiratory drive, making the calculation of Crs and Δ*P* during active breathing possible and accurate [16–20]. Secondly, driving pressure is the pressure dissipated against the elastic recoil of total respiratory system (Δ*P* = Δ*P*_chest wall_ plus Δ*P*_lung_), while it is well known that the injurious effects of high Δ*P* are related to high transpulmonary driving pressure (Δ*P*_lung_ = end-inspiratory minus end-expiratory transpulmonary pressure) [13, 21–23]. Although in our study driving transpulmonary pressures were not measured, the Δ*P* must always be higher than Δ*P*_lung_. As it has been shown that during passive mechanical ventilation a Δ*P* ≥ 15 cmH_2_O can detect lung overstress with an acceptable accuracy [24], it follows that, during PAV+, a Δ*P* below 15 cmH_2_O should be associated with low lung stress. Finally, the study entry criteria (estimated need for mechanical ventilation for at least 1 day after inclusion, and exclusion of patients requiring low levels of assist) resulted in a population of severely ill patients (APACHE-II score on admission 25). Most of the patients had ICU-acquired infections, and mild or moderate ARDS. Although patients were not formally identified as having difficult weaning, the prolonged duration of mechanical ventilation in this study group (median 18 days) should be acknowledged, emphasizing that the observed incidence of high Δ*P* is derived from a subset of critically ill patients with high severity scores and need for prolonged mechanical ventilation. Presumably, high driving pressure would be even rarer in patients with uncomplicated course and simple weaning.

In our previous study [15], 108 patients were switched from controlled mechanical ventilation to PAV+ and a median of eight measurements of Δ*P* per patient within 48 h of assisted mechanical ventilation was analyzed. These measurements showed that critically ill patients control their Δ*P* below 15 cmH_2_O by sizing *V*_T_ to individual respiratory system compliance. This is achieved by appropriate feedback systems (reflex: Hering–Breuer and chemical: ventilatory response to CO_2_). Indeed, it has been shown that, with proportional modes of support, these feedback mechanisms allow to maintain a safe range of tidal volume even at high assist [25, 26], since a decrease in patient effort through activation of chemical feedback and/or Hering reflex results in a proportional decrease in ventilator pressure. The current observational study, using continuous and prolonged measurements of Δ*P*, demonstrated that *V*_T_ and Δ*P* varied significantly over time. For brief periods of time (2.5 min), Δ*P* values ≥ 15 cmH_2_O occurred in many patients, but prolonged periods of high Δ*P* were observed in only 8% of patients. Although in these patients the contribution of chest wall to Δ*P* is not known, their clinical characteristics indicate that high Δ*P* is likely associated with high transpulmonary driving pressure. One patient had cryptogenic organizing pneumonia (COP) and another four had primary ARDS or decompensated congestive heart failure, conditions that decrease lung compliance and thus increase the contribution of transpulmonary pressure to Δ*P* values. Patients who died in the ICU overall had more time with Δ*P* above 15 cmH_2_O, yet, due to the small number of patients with prolonged high Δ*P*, no threshold of high Δ*P* duration associated with adverse outcome could be identified, and no causality could be established.

This study has some important clinical implications, which, however, should be evaluated in larger, randomized trials. Firstly, the incidence of high driving pressure, albeit small (8%), is not negligible, considering that these patients fulfilled criteria to be placed and maintained on assisted ventilation. Second, we showed that the presence of high Δ*P* was not associated with high tidal volume or high minute ventilation. The observed tidal volumes were in the range of 5–11 mL/kg, and not greater than 9.5 mL/kg during high Δ*P* periods. These results indicate that, in patients meeting criteria for assisted ventilation, the control of breathing mechanisms, chemical and reflex feedback mechanisms [27–29], often allows *V*_T_ to be higher than the recommended ‘safe’ range of 6–8 mL/kg. More importantly, high Δ*P* was strictly associated with low compliance; a threshold of 30 mL/cmH_2_O was identified, above which high Δ*P* is very rare. Additionally, Δ*P* was always high when compliance was below 20 mL/cmH_2_O and in half of the cases when compliance was below 25 mL/cmH_2_O. Therefore, provided that with conventional modes of support (assist volume control or pressure support) assist is not excessive, high Δ*P* is very unlikely to occur when respiratory system compliance is above 30 mL/cmH_2_O, even if *V*_T_ is higher than 8 mL/kg. On the other hand, while proportional modes such as PAV+ or neurally adjusted ventilatory assist (NAVA), are expected to provide a more protective ventilation [30], by allowing the operation of chemical and reflex feedback mechanisms [27–29, 31], this study indicates that when compliance is below 30 mL/cmH_2_O the protective mechanisms of control of breathing system may be overridden. Additionally, experimental and clinical data indicate that vigorous inspiratory efforts may promote lung injury, especially in the presence of severe underlying lung injury [9, 12, 13, 32]. Taken together, these findings suggest that when patients with lung injury and compliance below 30 mL/cmH_2_O are ventilated in assisted modes, they are at risk of developing high driving pressure, and physicians should consider monitoring driving or transpulmonary pressures.

This study has certain limitations that should be considered. The study included a group of patients with high disease severity scores, and prolonged mechanical ventilation, from a single center. Patients were studied whenever the primary physician placed them on PAV+, and not specifically when first placed in assisted mode. Moreover, chest wall mechanics were not evaluated, and thus, in some patients, high Δ*P* may not correspond to high transpulmonary pressure, due to low chest wall compliance. The driving pressure could also be overestimated in the presence of PEEPi (as PEEPi was not included in the calculation of compliance). In the population studied, the median PEEPi was low (0.3 cmH_2_O), and results were qualitatively the same when PEEPi was included in calculations. Most patients included in the study, as well as most patients admitted in the ICU, were overweight or obese, and patients with prolonged high driving pressure had even higher BMI. Finally, this study does not establish a causative relationship between high Δ*P* and mortality, but indicates that, given the small incidence of prolonged high Δ*P* identified, a very large study would be required to investigate this. Yet, this study identifies for the first time a safety threshold for respiratory system compliance during assisted ventilation at 30 mL/cmH_2_O, below which high driving pressures are more likely to occur. However, the clinical significance of this finding should be prospectively investigated.

## Conclusions

Continuous measurements of driving pressure in critically ill patients fulfilling criteria for assisted ventilation showed that sustained high driving pressure occurs, even in such patients, in 8% of cases. High driving pressure is not associated with high tidal volume or high minute ventilation, but with low compliance. A threshold of compliance greater than 30 mL/cmH_2_O was identified to exclude the presence of high driving pressure with 90% sensitivity and specificity.

## Additional files

**Additional file 1.** Methods.

**Additional file 2: Table S1.** Clinical and ventilation characteristics of patients with prolonged high Δ*P* ≥ 15 cmH_2_O.

## Abbreviations

APACHE-II: Acute Physiology And Chronic Health Evaluation II
ARDS: acute respiratory distress syndrome
COP: cryptogenic organizing pneumonia
Crs: respiratory system compliance
Δ*P*: driving pressure of respiratory system
ICU: intensive care unit
IQR: interquartile range
NAVA: neurally adjusted ventilatory assist
PAV+: proportional assist ventilation with load-adjustable gain factors
PEEP: positive end-expiratory pressure
Pplat: end-inspiratory plateau pressure
SD: standard deviation
VILI: ventilator-induced lung injury
*V*_T_: tidal volume
