# Supplementary material for: Driving pressure during proportional assist ventilation: an observational study
Source: Ann Intensive Care. 2019 Jan 3;9:1. doi: 10.1186/s13613-018-0477-4 (PMC6314935; doi:10.1186/s13613-018-0477-4)
Supplement: Supplementary file 1 — Additional file 1. Methods. [file 13613_2018_477_MOESM1_ESM.pdf]

Additional file 1

Driving Pressure during proportional assist ventilation: an observational study

Authors: Katerina Vaporidi MD, PhD[1], Charalambos Psarologakis MD[1], Athanasia Proklou MD, PhD [1], Emmanouil Pediaditis MD[1], Evangelia Akoumianaki MD, PhD [1], Elisavet Koutsiana[1,2], Achilleas Chytas[2,3], Ioanna Chouvarda PhD[2,3], Eumorfia Kondili MD, PhD [1], Dimitris Georgopoulos MD, PhD [1].

Affiliations:

[1]Department of Intensive Care Medicine, University Hospital of Heraklion and School of Medicine, University of Crete, Heraklion, Greece

[2]Lab of Computing Medical Informatics and Biomedical Imaging Technologies, School of Medicine, Aristotle University of Thessaloniki, Thessaloniki, Greece.

[3]Institute of Applied Biosciences, CERTH, Thessaloniki, Greece.

Methods

Data Collection

Inclusion and exclusion criteria: patients were studied at any time the treating physician placed them in PAV+ mode and estimated that they will remain on assisted mechanical ventilation for more than one day (in this 12-bed ICU there are five ventilators providing PAV+ mode, and thus the decision to ventilate a patient on PAV+ depended not only on physician's preference but also on each patient's bedside ventilator). Patients were excluded if the level of assist in PAV+, as chosen by the primary physician, was less than 20%, since under these conditions airway pressure measured at the end of the 0.3 sec occlusion (Pplat) may be underestimated (1). Patients were also excluded when the necessary equipment (connecting cables and bedside computer) for the recording were not available. Additionally, patients with chest tubes inserted, and patients with obstructive lung disease and presence of intrinsic PEEP in control modes were not included, as such patients are not placed on PAV+ when switched to assisted modes.

The ventilatory protocol in PAV+ used in this study has been previously described [3]. Briefly, an initial assist level of 50% was applied, and titrated to achieve patient comfort, respiratory rate below 35 br/min and tidal volume 5-8 ml/kg. The recording period was 72 hours, unless the patient was placed on T-piece earlier. If the patient was switched to other modes of ventilation the recording was not interrupted, but this time was excluded from analysis. The treating physicians could change ventilator mode during the recording at their

best judgement. The ventilator was connected to the bedside computer and a continuous recording of all ventilator parameters was obtained at a frequency of 0.8 Hz using an appropriate software (Puritan-Bennett, Covidien, MN, USA), and a script to obtain continuous measurements. All files were stored for later analysis.

Data analysis

Compliance and Driving Pressure measurements

The recordings were processed before analysis to 1) optimize data quality (e.g. rejection of artifacts) and 2) exclude the measurements obtained in other modes of ventilation. For this purpose, a dedicated software was developed using R programming language and software environment. Driving pressure (ΔP) was calculated from the measurements of respiratory system compliance (Crs) and tidal volume (V_T) as $\Delta P = V_T/Crs$ (Additional file Figure).

In PAV+ the ventilator's software calculates the respiratory system resistance (Rrs) and Crs as described in detail by Younes et al. (1, 2). Briefly, for the calculation of compliance the ventilator randomly applies a 0.3-sec end-inspiratory occlusion every 4-7 breaths and measures airway pressure at the end of occlusion (Pplat). The scientific reasoning behind the calculation of Crs is described below:

With PAV+ at time t from the beginning of inspiration, inspiratory muscle pressure ($Pmus_{I(t)}$) is given by the following equation:

$Pmus_{I(t)}=V_{(t)}*(Ers-\%assist*Ers/100)+V'_{(t)}*(Rrs-\%assist*Rrs/100)$ [Eq. 1],

where $V_{(t)}$ and $V'_{(t)}$ are inspiratory volume and flow at time t from the beginning of inspiration, respectively and Ers is elastance of respiratory system (1/Crs). During the rising phase of $Pmus_I$, $Pmus_I$ is always greater than the term $V_{(t)}$*(Ers-%assist*Ers/100) and thus inspiratory flow is produced until peak $Pmus_I$ is achieved ($Pmus_{Ipeak}$). $Pmus_{Ipeak}$ is given by:

$Pmus_{Ipeak}$=V_{peak}*(Ers–%assist*Ers/100)+V'_{peak}*(Rrs-%assist*Rrs/100) [Eq. 2],

where V_{peak} and V'_{peak} are inspiratory volume and flow at peak Pmus, respectively.

Thereafter $Pmus_I$ declines and inspiratory flow continues, although in decreasing amounts. On the other hand, since there is inspiratory flow the volume continues to rise, increasing the volume term $V_{(t)}$*(Ers-%assistxErs). When $Pmus_I$ is equal to the volume term there is no pressure available for flow and flow becomes expiratory flow driven by the elastic recoil pressure. In breaths selected for occlusion maneuver, an occlusion for 0.3 sec is applied when inspiratory flow reaches a value of 0.05 l/sec, (i.e. close to zero). The $Pmus_I$ at the beginning of occlusion (zero flow) is:

$Pmus_I$=V_T*(Ers-%assist*Ers/100) [Eq. 3],

where V_T is tidal volume.

Eq. 3 dictates that the remaining $Pmus_I$ at the beginning of occlusion is low when V_T is low and/or the % of assist is high. It has been shown that independent of respiratory drive, at the end of occlusion time (0.3 sec) $Pmus_I$ has declined to zero or close to zero, even if the rate of decline is very slow (i.e. 5 cmH_2O/sec). Therefore, Paw at 0.3 sec of occlusion is similar to passive elastic recoil pressure corresponding to V_T of the occluded breath. Assuming that there is no dynamic hyperinflation and the pressure-volume

relationship is linear at the range of V_T, respiratory system compliance is calculated as:

$$Crs = V_T/(Pplat\text{-}PEEP) \text{ [Eq. 4]},$$

where PEEP is positive end-expiratory pressure.

Solving for Pplat-PEEP (driving pressure, ΔP)

$$\Delta P = Plat\text{-}PEEP = V_T/Crs \text{ [Eq. 5]}.$$

Although Crs may not be constant during the range of observed tidal volumes due to non-linear pressure-volume relationships, the measurement of Pplat represents the true passive elastic recoil pressure at the end of inspiration. Thus, independent of pressure-volume relationship, the calculation of the driving pressure is a valid estimate of the change in elastic pressure due to V_T. Data from animal and human studies have shown that expiratory muscle activity, if any begins well after the release of occlusion (1, 2). The time course of elastic recoil pressure early in expiration is used (since Crs is known) to estimate the driving pressure for flow and Rrs is calculated as the ratio of this pressure by the corresponding flow (2).

Intrinsic PEEP (PEEPi) is estimated by the ventilator software using the following technique. Since Rrs and Ers have been measured, the software, assuming that expiration is passive, estimates alveolar pressure (Palv) from the beginning to the end of expiration. $PEEP_i$ is calculated as the difference (if it exists) between Palv and PEEP at the end of expiration. Ventilator software does not know the exact expiration end time and consequently searches for two events during exhalation. The first occurs when Palv > Paw. At this moment, the ventilator captures and stores the values of Palv 100 ms earlier and identifies this value for the estimation of $PEEP_i$ if a breath is triggered; in

this case, by definition $PEEP_i$ = Palv – PEEP. However, if no breath is triggered, this earlier estimate of Palv is abandoned. Each successive event for which Palv > Paw is treated in the same way. The second event occurs when expiratory flow becomes zero before triggering. If expiratory flow becomes zero before a breath is triggered, then Palv = Paw = PEEP and thus Palv – PEEP = 0 (i.e. $PEEP_i$=0).

For safety purposes the ventilator software subjects all measurements to checks, and the estimates of Crs, and thus of Rrs, are discarded if any of the rejection pre-defined criteria are met (1, 2). Although ΔP is the measured Pplat minus PEEP, the ventilator software used during the study did not provide the direct measurement of Pplat, and thus the calculated compliance is used to compute ΔP. Ventilator's software also measures PEEPi (1, 2, see above), but for the purpose of this study and for the calculation of ΔP, PEEPi was not included in equation. We assume that due to the low levels of PEEPi measured in our patients (median 0.3, IQR=0.1-0.7) and the absence of patients with acute exacerbation of obstructive lung disease, the contribution of PEEPi to ΔP would be negligible. Nevertheless, ΔP without taking into consideration the presence of PEEPi, if any, is overestimated. It should be noted also that during control mechanical ventilation Pplat is usually measured at the end of a 3-sec occlusion (3-5), and, as the duration of inspiratory pause affects the measured plateau pressure (6, 7), due to stress relaxation and time-constant inequalities of the respiratory system (8), all else being the same, ΔP during PAV+ would be higher than that during passive mechanical ventilation by the change in Pplat between 0.3 sec and 3.0 sec after occlusion.

Analysis of ventilator output data

Three types of analysis were performed

1) The measured V_T and computed ΔP values, after quality optimization (rejection of artifacts and measurements obtained in modes other than PAV+), were separated in single unit intervals from less than 5 to more than 15 cmH_2O, and the percentage of time values within each time range was calculated. These data were analyzed without smoothing of ΔP measurements.

2) Periods of high ΔP sustained for more than one hour were identified after applying a smoothing to the ΔP signal, using the moving median method and an 11-size window (Figure 2). A time frame of at least one hour was chosen so that possible correlations with the hourly collected data on vital signs and medication infusions could be explored. The following clinical parameters were examined and characterized as present or absent during the high-ΔP period and the rest of the recorded period: fever (>38.1), metabolic acidosis, delirium (positive if +CAM-ICU score or administration of haloperidol), sedation, opioid analgesia, and shock.

3) Periods of stable compliance were identified after analyzing the slope of the Crs signal (Figure 2). This analysis was performed to evaluate ventilator variables at different levels of compliance, as compliance is the independent variable during ventilation. A linear segmentation method was used, to locate the slope change points in the time series, with a window size of 500 samples, and a slope change (as angle) of 150 degrees (the maximum angle in degrees that the running average of the slopes in the current set of

points must change relative to the slope of the data calculated in the most current window before a change-point is recorded). The slope value of each part in the time series between two slope-change points was calculated. Parts were characterized as stable when the slope value was between -0.001 and 0.001.

For the complete analyzed period, and for all selected periods, the mean, median, standard deviation, and interquartile range for all set and measured parameters of the ventilator were calculated using R programming language and software environment. To evaluate the response of the various ventilatory variables to changes in compliance, in every patient two sequential periods (period 1 and period 2) of stable compliance were used to compute their differences (value during period 2 minus value during period 1). Obviously, the number of stable Crs periods was not the same in all patients. Only two patients did not have two stable periods to permit comparisons, thus these two patients were not included in this type of analysis.

References

1. Younes M, Webster K, Kun J, Roberts D, Masiowski B. A method for measuring passive elastance during proportional assist ventilation. American journal of respiratory and critical care medicine. 2001;164(1):50-60.

2. Younes M, Kun J, Masiowski B, Webster K, Roberts D. A method for noninvasive determination of inspiratory resistance during proportional assist ventilation. American journal of respiratory and critical care medicine. 2001;163(4):829-39.

3. Laffey JG, Bellani G, Pham T, Fan E, Madotto F, Bajwa EK, et al. Potentially modifiable factors contributing to outcome from acute respiratory distress syndrome: the LUNG SAFE study. Intensive care medicine. 2016;42(12):1865-76.

4. Amato MB, Meade MO, Slutsky AS, Brochard L, Costa EL, Schoenfeld DA, et al. Driving pressure and survival in the acute respiratory distress syndrome. The New England journal of medicine. 2015;372(8):747-55.

5. Bellani G, Grasselli G, Teggia-Droghi M, Mauri T, Coppadoro A, Brochard L, et al. Do spontaneous and mechanical breathing have similar effects on average transpulmonary and alveolar pressure? A clinical crossover study. Critical care. 2016;20(1):142.

6. Barberis L, Manno E, Guerin C. Effect of end-inspiratory pause duration on plateau pressure in mechanically ventilated patients. Intensive care medicine. 2003;29(1):130-4.

7. Mezidi M, Yonis H, Aublanc M, Lissonde F, Louf-Durier A, Perinel S, et al. Effect of end-inspiratory plateau pressure duration on driving pressure. Intensive care medicine. 2017;43(4):587-9.

8. Bates JH, Rossi A, Milic-Emili J. Analysis of the behavior of the respiratory system with constant inspiratory flow. Journal of applied physiology. 1985;58(6):1840-8.

Figure Legend:

Method of calculation of respiratory system compliance in PAV+

Method of calculation of driving pressure (ΔP) and respiratory system compliance (Crs) in PAV+ in comparison to volume control mode ($Crs=V_T/\Delta P$). Tracings of airway, esophageal, and transpulmonary pressures are shown in upper panels, and flow in lower panels, in volume control mode (left), and PAV+ (right). The grey shaded area indicates the period of zero flow, and the horizontal dotted line the pressure at zero flow, used for the calculation of driving pressure. The driving pressure of the respiratory system (ΔP) represents the difference, at zero flow conditions, between end-inspiratory (plateau) airway pressure and total positive end expiratory pressure, while the transpulmonary driving pressure (ΔP_L) represents the difference between the corresponding end-inspiratory and end-expiratory transpulmonary pressures. The difference between ΔP and ΔP_L represents the driving pressure of the chest wall ($\Delta P = \Delta P_L + \Delta Pcw$). The measurement of ΔPrs in volume control mode is performed at the end of a 3-sec manual inspiratory pause, and in PAV+ at the end of an automated 0.3-sec inspiratory pause.

Additional file 1

Driving Pressure during proportional assist ventilation: an observational study

Authors: Katerina Vaporidi MD, PhD[1], Charalambos Psarologakis MD[1], Athanasia Proklou MD, PhD [1], Emmanouil Pediaditis MD[1], Evangelia Akoumianaki MD, PhD [1], Elisavet Koutsiana[1,2], Achilleas Chytas[2,3], Ioanna Chouvarda PhD[2,3], Eumorfia Kondili MD, PhD [1], Dimitris Georgopoulos MD, PhD [1].

Affiliations:

[1]Department of Intensive Care Medicine, University Hospital of Heraklion and School of Medicine, University of Crete, Heraklion, Greece

[2]Lab of Computing Medical Informatics and Biomedical Imaging Technologies, School of Medicine, Aristotle University of Thessaloniki, Thessaloniki, Greece.

[3]Institute of Applied Biosciences, CERTH, Thessaloniki, Greece.

Methods

Data Collection

Inclusion and exclusion criteria: patients were studied at any time the treating physician placed them in PAV+ mode and estimated that they will remain on assisted mechanical ventilation for more than one day (in this 12-bed ICU there are five ventilators providing PAV+ mode, and thus the decision to ventilate a patient on PAV+ depended not only on physician's preference but also on each patient's bedside ventilator). Patients were excluded if the level of assist in PAV+, as chosen by the primary physician, was less than 20%, since under these conditions airway pressure measured at the end of the 0.3 sec occlusion (Pplat) may be underestimated (1). Patients were also excluded when the necessary equipment (connecting cables and bedside computer) for the recording were not available. Additionally, patients with chest tubes inserted, and patients with obstructive lung disease and presence of intrinsic PEEP in control modes were not included, as such patients are not placed on PAV+ when switched to assisted modes.

The ventilatory protocol in PAV+ used in this study has been previously described [3]. Briefly, an initial assist level of 50% was applied, and titrated to achieve patient comfort, respiratory rate below 35 br/min and tidal volume 5-8 ml/kg. The recording period was 72 hours, unless the patient was placed on T-piece earlier. If the patient was switched to other modes of ventilation the recording was not interrupted, but this time was excluded from analysis. The treating physicians could change ventilator mode during the recording at their

best judgement. The ventilator was connected to the bedside computer and a continuous recording of all ventilator parameters was obtained at a frequency of 0.8 Hz using an appropriate software (Puritan-Bennett, Covidien, MN, USA), and a script to obtain continuous measurements. All files were stored for later analysis.

Data analysis

Compliance and Driving Pressure measurements

The recordings were processed before analysis to 1) optimize data quality (e.g. rejection of artifacts) and 2) exclude the measurements obtained in other modes of ventilation. For this purpose, a dedicated software was developed using R programming language and software environment. Driving pressure (ΔP) was calculated from the measurements of respiratory system compliance (Crs) and tidal volume (V_T) as $\Delta P = V_T/Crs$ (Additional file Figure).

In PAV+ the ventilator's software calculates the respiratory system resistance (Rrs) and Crs as described in detail by Younes et al. (1, 2). Briefly, for the calculation of compliance the ventilator randomly applies a 0.3-sec end-inspiratory occlusion every 4-7 breaths and measures airway pressure at the end of occlusion (Pplat). The scientific reasoning behind the calculation of Crs is described below:

With PAV+ at time t from the beginning of inspiration, inspiratory muscle pressure ($Pmus_{I(t)}$) is given by the following equation:

$Pmus_{I(t)}=V_{(t)}*(Ers-\%assist*Ers/100)+V'_{(t)}*(Rrs-\%assist*Rrs/100)$ [Eq. 1],

where $V_{(t)}$ and $V'_{(t)}$ are inspiratory volume and flow at time t from the beginning of inspiration, respectively and Ers is elastance of respiratory system (1/Crs).

During the rising phase of $Pmus_I$, $Pmus_I$ is always greater than the term $V_{(t)}$*(Ers-%assist*Ers/100) and thus inspiratory flow is produced until peak $Pmus_I$ is achieved ($Pmus_{Ipeak}$). $Pmus_{Ipeak}$ is given by:

$Pmus_{Ipeak}=V_{peak}$*(Ers–%assist*Ers/100)+V'_{peak}*(Rrs-%assist*Rrs/100) [Eq. 2],

where V_{peak} and V'_{peak} are inspiratory volume and flow at peak Pmus, respectively.

Thereafter $Pmus_I$ declines and inspiratory flow continues, although in decreasing amounts. On the other hand, since there is inspiratory flow the volume continues to rise, increasing the volume term $V_{(t)}$*(Ers-%assistxErs). When $Pmus_I$ is equal to the volume term there is no pressure available for flow and flow becomes expiratory flow driven by the elastic recoil pressure. In breaths selected for occlusion maneuver, an occlusion for 0.3 sec is applied when inspiratory flow reaches a value of 0.05 l/sec, (i.e. close to zero). The $Pmus_I$ at the beginning of occlusion (zero flow) is:

$Pmus_I=V_T$*(Ers-%assist*Ers/100) [Eq. 3],

where V_T is tidal volume.

Eq. 3 dictates that the remaining $Pmus_I$ at the beginning of occlusion is low when V_T is low and/or the % of assist is high. It has been shown that independent of respiratory drive, at the end of occlusion time (0.3 sec) $Pmus_I$ has declined to zero or close to zero, even if the rate of decline is very slow (i.e. 5 cmH_2O/sec). Therefore, Paw at 0.3 sec of occlusion is similar to passive elastic recoil pressure corresponding to V_T of the occluded breath. Assuming that there is no dynamic hyperinflation and the pressure-volume

relationship is linear at the range of V_T, respiratory system compliance is calculated as:

$$Crs = V_T/(Pplat\text{-}PEEP) \text{ [Eq. 4]},$$

where PEEP is positive end-expiratory pressure.

Solving for Pplat-PEEP (driving pressure, ΔP)

$$\Delta P = Plat\text{-}PEEP = V_T/Crs \text{ [Eq. 5]}.$$

Although Crs may not be constant during the range of observed tidal volumes due to non-linear pressure-volume relationships, the measurement of Pplat represents the true passive elastic recoil pressure at the end of inspiration. Thus, independent of pressure-volume relationship, the calculation of the driving pressure is a valid estimate of the change in elastic pressure due to V_T. Data from animal and human studies have shown that expiratory muscle activity, if any begins well after the release of occlusion (1, 2). The time course of elastic recoil pressure early in expiration is used (since Crs is known) to estimate the driving pressure for flow and Rrs is calculated as the ratio of this pressure by the corresponding flow (2).

Intrinsic PEEP (PEEPi) is estimated by the ventilator software using the following technique. Since Rrs and Ers have been measured, the software, assuming that expiration is passive, estimates alveolar pressure (Palv) from the beginning to the end of expiration. $PEEP_i$ is calculated as the difference (if it exists) between Palv and PEEP at the end of expiration. Ventilator software does not know the exact expiration end time and consequently searches for two events during exhalation. The first occurs when Palv > Paw. At this moment, the ventilator captures and stores the values of Palv 100 ms earlier and identifies this value for the estimation of $PEEP_i$ if a breath is triggered; in

this case, by definition $PEEP_i$ = Palv – PEEP. However, if no breath is triggered, this earlier estimate of Palv is abandoned. Each successive event for which Palv > Paw is treated in the same way. The second event occurs when expiratory flow becomes zero before triggering. If expiratory flow becomes zero before a breath is triggered, then Palv = Paw = PEEP and thus Palv – PEEP = 0 (i.e. $PEEP_i$=0).

For safety purposes the ventilator software subjects all measurements to checks, and the estimates of Crs, and thus of Rrs, are discarded if any of the rejection pre-defined criteria are met (1, 2). Although ΔP is the measured Pplat minus PEEP, the ventilator software used during the study did not provide the direct measurement of Pplat, and thus the calculated compliance is used to compute ΔP. Ventilator's software also measures PEEPi (1, 2, see above), but for the purpose of this study and for the calculation of ΔP, PEEPi was not included in equation. We assume that due to the low levels of PEEPi measured in our patients (median 0.3, IQR=0.1-0.7) and the absence of patients with acute exacerbation of obstructive lung disease, the contribution of PEEPi to ΔP would be negligible. Nevertheless, ΔP without taking into consideration the presence of PEEPi, if any, is overestimated. It should be noted also that during control mechanical ventilation Pplat is usually measured at the end of a 3-sec occlusion (3-5), and, as the duration of inspiratory pause affects the measured plateau pressure (6, 7), due to stress relaxation and time-constant inequalities of the respiratory system (8), all else being the same, ΔP during PAV+ would be higher than that during passive mechanical ventilation by the change in Pplat between 0.3 sec and 3.0 sec after occlusion.

Analysis of ventilator output data

Three types of analysis were performed

1) The measured V_T and computed ΔP values, after quality optimization (rejection of artifacts and measurements obtained in modes other than PAV+), were separated in single unit intervals from less than 5 to more than 15 cmH_2O, and the percentage of time values within each time range was calculated. These data were analyzed without smoothing of ΔP measurements.

2) Periods of high ΔP sustained for more than one hour were identified after applying a smoothing to the ΔP signal, using the moving median method and an 11-size window (Figure 2). A time frame of at least one hour was chosen so that possible correlations with the hourly collected data on vital signs and medication infusions could be explored. The following clinical parameters were examined and characterized as present or absent during the high-ΔP period and the rest of the recorded period: fever (>38.1), metabolic acidosis, delirium (positive if +CAM-ICU score or administration of haloperidol), sedation, opioid analgesia, and shock.

3) Periods of stable compliance were identified after analyzing the slope of the Crs signal (Figure 2). This analysis was performed to evaluate ventilator variables at different levels of compliance, as compliance is the independent variable during ventilation. A linear segmentation method was used, to locate the slope change points in the time series, with a window size of 500 samples, and a slope change (as angle) of 150 degrees (the maximum angle in degrees that the running average of the slopes in the current set of

points must change relative to the slope of the data calculated in the most current window before a change-point is recorded). The slope value of each part in the time series between two slope-change points was calculated. Parts were characterized as stable when the slope value was between -0.001 and 0.001.

For the complete analyzed period, and for all selected periods, the mean, median, standard deviation, and interquartile range for all set and measured parameters of the ventilator were calculated using R programming language and software environment. To evaluate the response of the various ventilatory variables to changes in compliance, in every patient two sequential periods (period 1 and period 2) of stable compliance were used to compute their differences (value during period 2 minus value during period 1). Obviously, the number of stable Crs periods was not the same in all patients. Only two patients did not have two stable periods to permit comparisons, thus these two patients were not included in this type of analysis.

References

1. Younes M, Webster K, Kun J, Roberts D, Masiowski B. A method for measuring passive elastance during proportional assist ventilation. American journal of respiratory and critical care medicine. 2001;164(1):50-60.

2. Younes M, Kun J, Masiowski B, Webster K, Roberts D. A method for noninvasive determination of inspiratory resistance during proportional assist ventilation. American journal of respiratory and critical care medicine. 2001;163(4):829-39.

3. Laffey JG, Bellani G, Pham T, Fan E, Madotto F, Bajwa EK, et al. Potentially modifiable factors contributing to outcome from acute respiratory distress syndrome: the LUNG SAFE study. Intensive care medicine. 2016;42(12):1865-76.

4. Amato MB, Meade MO, Slutsky AS, Brochard L, Costa EL, Schoenfeld DA, et al. Driving pressure and survival in the acute respiratory distress syndrome. The New England journal of medicine. 2015;372(8):747-55.

5. Bellani G, Grasselli G, Teggia-Droghi M, Mauri T, Coppadoro A, Brochard L, et al. Do spontaneous and mechanical breathing have similar effects on average transpulmonary and alveolar pressure? A clinical crossover study. Critical care. 2016;20(1):142.

6. Barberis L, Manno E, Guerin C. Effect of end-inspiratory pause duration on plateau pressure in mechanically ventilated patients. Intensive care medicine. 2003;29(1):130-4.

7. Mezidi M, Yonis H, Aublanc M, Lissonde F, Louf-Durier A, Perinel S, et al. Effect of end-inspiratory plateau pressure duration on driving pressure. Intensive care medicine. 2017;43(4):587-9.

8. Bates JH, Rossi A, Milic-Emili J. Analysis of the behavior of the respiratory system with constant inspiratory flow. Journal of applied physiology. 1985;58(6):1840-8.

Figure: Method of calculation of respiratory system compliance in PAV+

Method of calculation of driving pressure (ΔP) and respiratory system compliance (Crs) in PAV+ in comparison to volume control mode ($Crs=V_T/\Delta P$). Tracings of airway, esophageal, and transpulmonary pressures are shown in upper panels, and flow in lower panels, in volume control mode (left), and PAV+ (right). The grey shaded area indicates the period of zero flow, and the horizontal dotted line the pressure at zero flow, used for the calculation of driving pressure. The driving pressure of the respiratory system (ΔP) represents the difference, at zero flow conditions, between end-inspiratory (plateau) airway pressure and total positive end expiratory pressure, while the transpulmonary driving pressure (ΔP_L) represents the difference between the corresponding end-inspiratory and end-expiratory transpulmonary pressures. The difference between ΔP and ΔP_L represents the driving pressure of the chest wall ($\Delta P = \Delta P_L + \Delta Pcw$). The measurement of ΔPrs in volume control mode is performed at the end of a 3-sec manual inspiratory pause, and in PAV+ at the end of an automated 0.3-sec inspiratory pause.

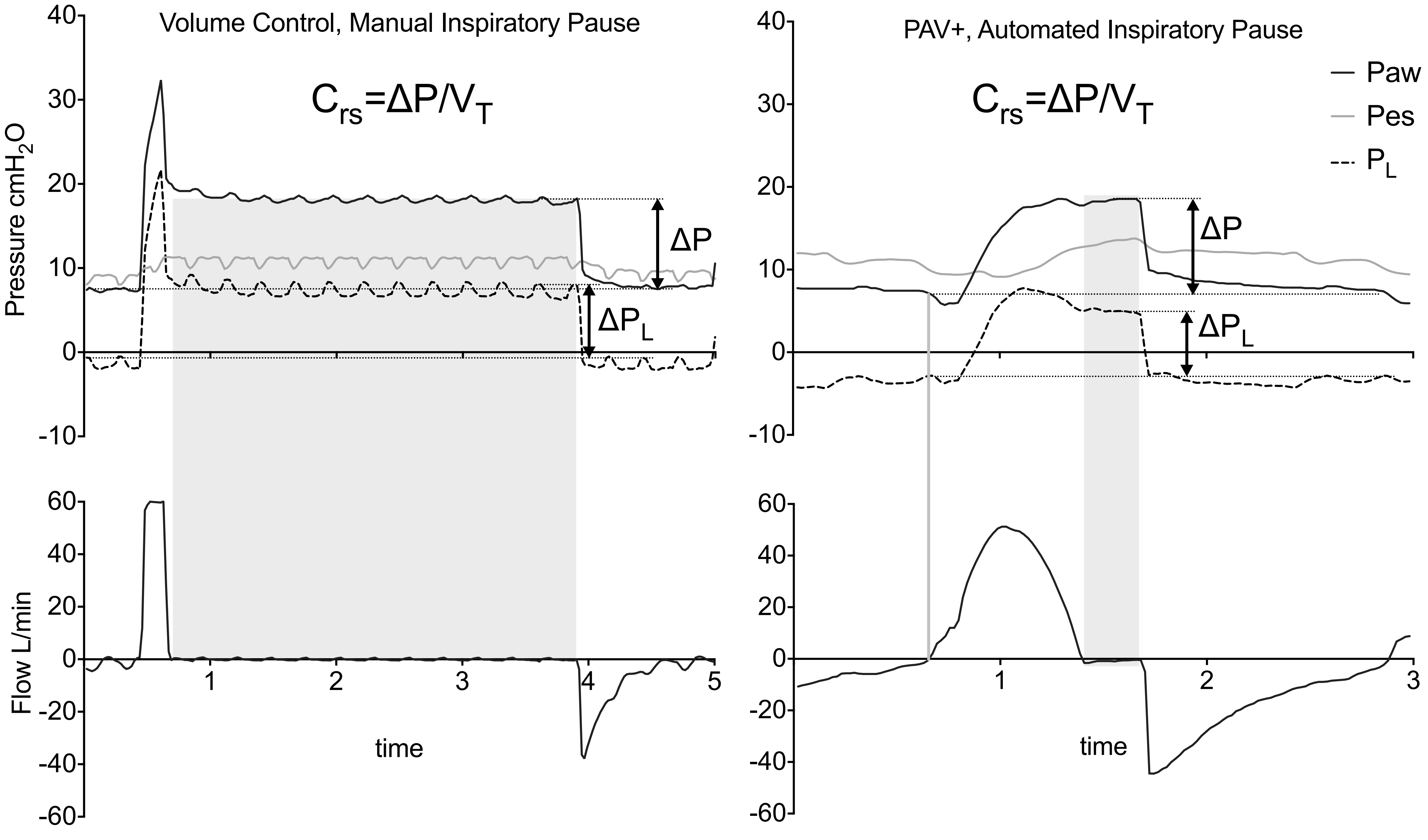

Volume Control, Manual Inspiratory Pause
PAV+, Automated Inspiratory Pause
$C_{rs}=\Delta P/V_T$
$C_{rs}=\Delta P/V_T$
Paw
Pes
P_L
ΔP
ΔP_L
ΔP
ΔP_L
Pressure cmH_2O
Flow L/min
time
time
