# Supplementary material for: Driving pressure during proportional assist ventilation: an observational study
Source: Ann Intensive Care. 2019 Jan 3;9:1. doi: 10.1186/s13613-018-0477-4 (PMC6314935; doi:10.1186/s13613-018-0477-4)
Supplement: Supplementary file 2 — Additional file 2: Table S1. Clinical and ventilation characteristics of patients with prolonged high ΔP ≥ 15 cmH2O. [file 13613_2018_477_MOESM2_ESM.docx]

Table S1: Clinical and ventilation characteristics of patients with prolonged high ΔP ≥ 15 cmH_2_O

| Patient | Age | Sex | BMI | SOFA  Day 1 | APACHE-II Day 1 | Diagnosis | PO_2_/  FiO_2_^1^ | V_T_^2^ ml/kg | V_T_^3^ ml/kg | Crs^2^ | Crs^3^ | Time with high ΔP^4^ hrs (%) | Outcome |
| --- | --- | --- | --- | --- | --- | --- | --- | --- | --- | --- | --- | --- | --- |
| 1 | 71 | F | 39 | 7 | 17 | Respiratory Failure, COP | 135 | 7.9 | 8.0 | 31 | 24 | 19.9 (71) | dead |
| 2 | 75 | F | 47 | 9 | 29 | Sepsis, decompensated CHF | 135 | 5.6 | 5.2 | 32 | 18 | 12.5 (40) | dead |
| 3 | 60 | F | 33 | 8 | 24 | ARDS secondary, sepsis | 200 | 7.7 | 7.3 | 71 | 30 | 29.5 (42) | alive |
| 4 | 48 | F | 27 | 12 | 11 | Upper GI bleeding, ARDS (VAP) | 230 | 6.5 | 6.8 | 34 | 28 | 13.7 (17) | alive |
| 5 | 76 | F | 36 | 5 | 19 | Hemorrhagic shock -trauma, decompensated CHF | 143 | 7.1 | 9.2 | 28 | 24 | 36.8 (41) | dead |

^1^ PO_2_/FiO_2_ at the time of initiation of recording; ^2^ V_T_ ml/kg IBW: median tidal volume for the entire analyzed period; ^3^ V_T_ ml/kg IBW: median tidal volume for the period of high ΔP; ^2^ Crs (ml/cmH_2_O): median compliance for the entire analyzed period; ^3^ Crs (ml/cmH_2_O): median compliance during the period of high ΔP; ^4^ Total time with high ΔP ≥15 cmH_2_O, as identified without smoothing of ΔP signal, in hours and as % of the total analyzed time.

Abbreviations:

BMI: body mass index; SOFA: Sequential Organ Failure Assessment (severity score); APACHE-II: Acute Physiology and Chronic Health Evaluation II (severity score); PO_2_/FiO_2_: ratio of arterial oxygen partial pressure to fractional inspired oxygen; Crs: respiratory system compliance; ΔP: driving pressure; M, F: male, female; COP: cryptogenic organizing pneumonia; CHF: congestive heart failure; ARDS: acute respiratory distress syndrome (Berlin definition); GI: gastrointestinal; VAP: ventilator-associated pneumonia.
